# Supplementary material for: Disentangling crossing fibers with advanced dMRI methods reveals bundle-specific degeneration across the visual system in asymmetric glaucoma
Source: PLoS One. 2026 Jun 22;21(6):e0349951. doi: 10.1371/journal.pone.0349951 (PMC13286229; doi:10.1371/journal.pone.0349951)
Supplement: S1 Fig — Raw and preprocessed images and derived maps are shown. The first two columns show the average images at b = 0 and b = 2500 s/mm², respectively, followed by FA and principal diffusivity direction color-coded maps (V1) with boxes indicating the magnified regions in the rightmost column. Even before preprocessing, the MUSE acquisition shows overall better image quality and improved maps of diffusion metrics. The region of fiber decussation at the level of chiasm is better observed in the MUSE acquisition as a red region indicating fibers crossing the midline (long arrow in the enlarged V1 map). Contrarily, the HB acquisition shows considerable signal pile-up (yellow arrowheads) that result in inadequate estimation of diffusion metrics within the region that corresponds to the optic chiasm and tracts (white arrowheads in enlarged V1). While these artifacts are minimized after preprocessing the HB acquisition, the decussation of fibers is mostly missing. (PDF) [file pone.0349951.s001.pdf]

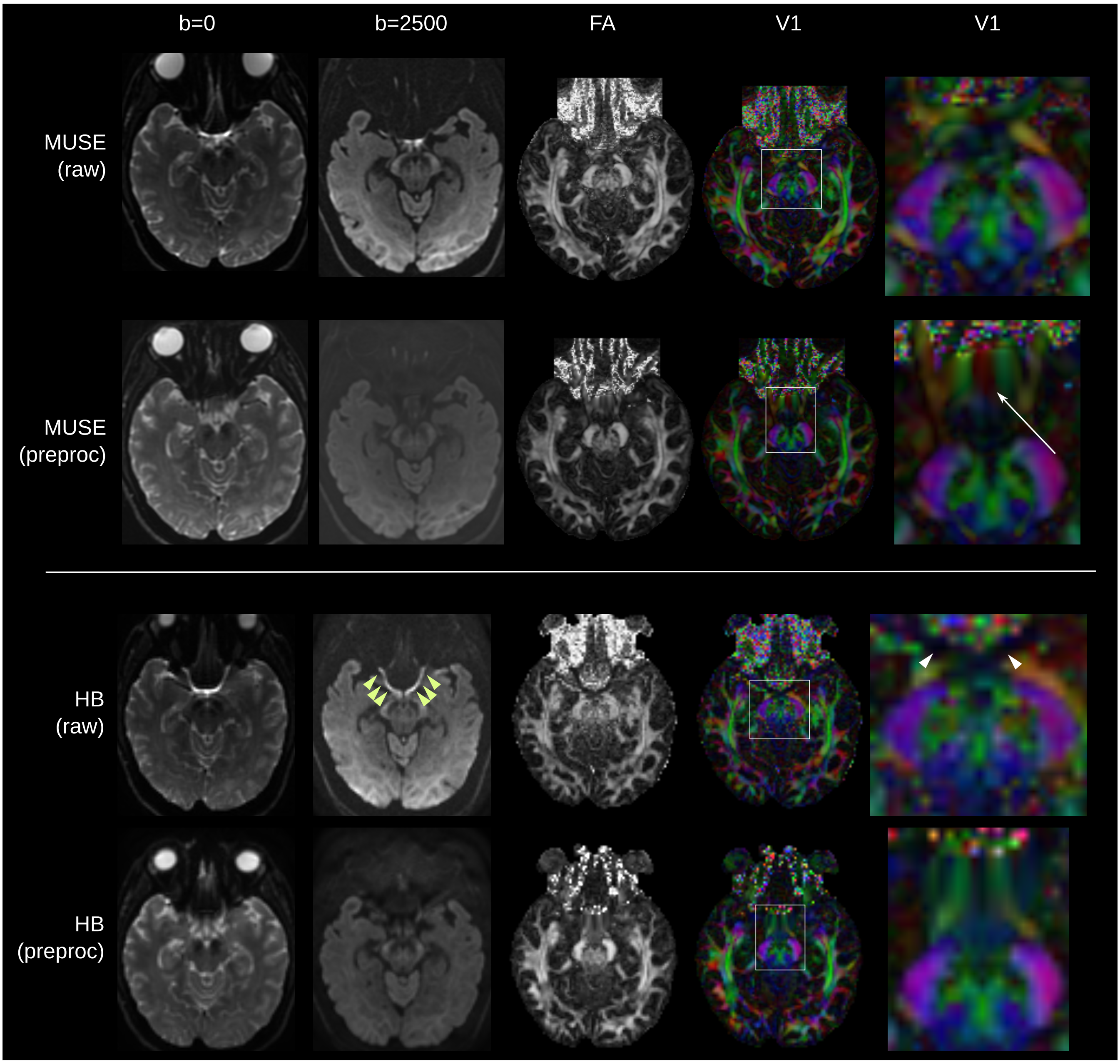

Supplementary Fig S1: Comparison of MUSE and Hyperband (HB) acquisitions. Raw and preprocessed images and derived maps are shown. The first two columns show the average images at b=0 and b=2500 s/mm², respectively, followed by FA and principal diffusivity direction color-coded maps (V1) with boxes indicating the magnified regions in the rightmost column. Even before preprocessing, the MUSE acquisition shows overall better image quality and improved maps of diffusion metrics. The region of fiber decussation at the level of chiasm is better observed in the MUSE acquisition as a red region indicating fibers crossing the midline (long arrow in the enlarged V1 map). Contrarily, the HB acquisition shows considerable signal pile-up (yellow arrowheads) that result in inadequate estimation of diffusion metrics within the region that corresponds to the optic chiasm and tracts (white arrowheads in enlarged V1). While these artifacts are minimized after preprocessing the HB acquisition, the decussation of fibers is mostly missing
