# Supplementary material for: Disentangling crossing fibers with advanced dMRI methods reveals bundle-specific degeneration across the visual system in asymmetric glaucoma
Source: PLoS One. 2026 Jun 22;21(6):e0349951. doi: 10.1371/journal.pone.0349951 (PMC13286229; doi:10.1371/journal.pone.0349951)
Supplement: S2 Fig — Significant correlations are shown as circles (blue = negative, red = positive). Circle diameters reflect the significance of the correlation (p < 0.05), and crosses indicate trends (0.05 < p < 0.1). The average diffusion metrics of both hemispheres is used for the optic radiations. (PDF) [file pone.0349951.s002.pdf]

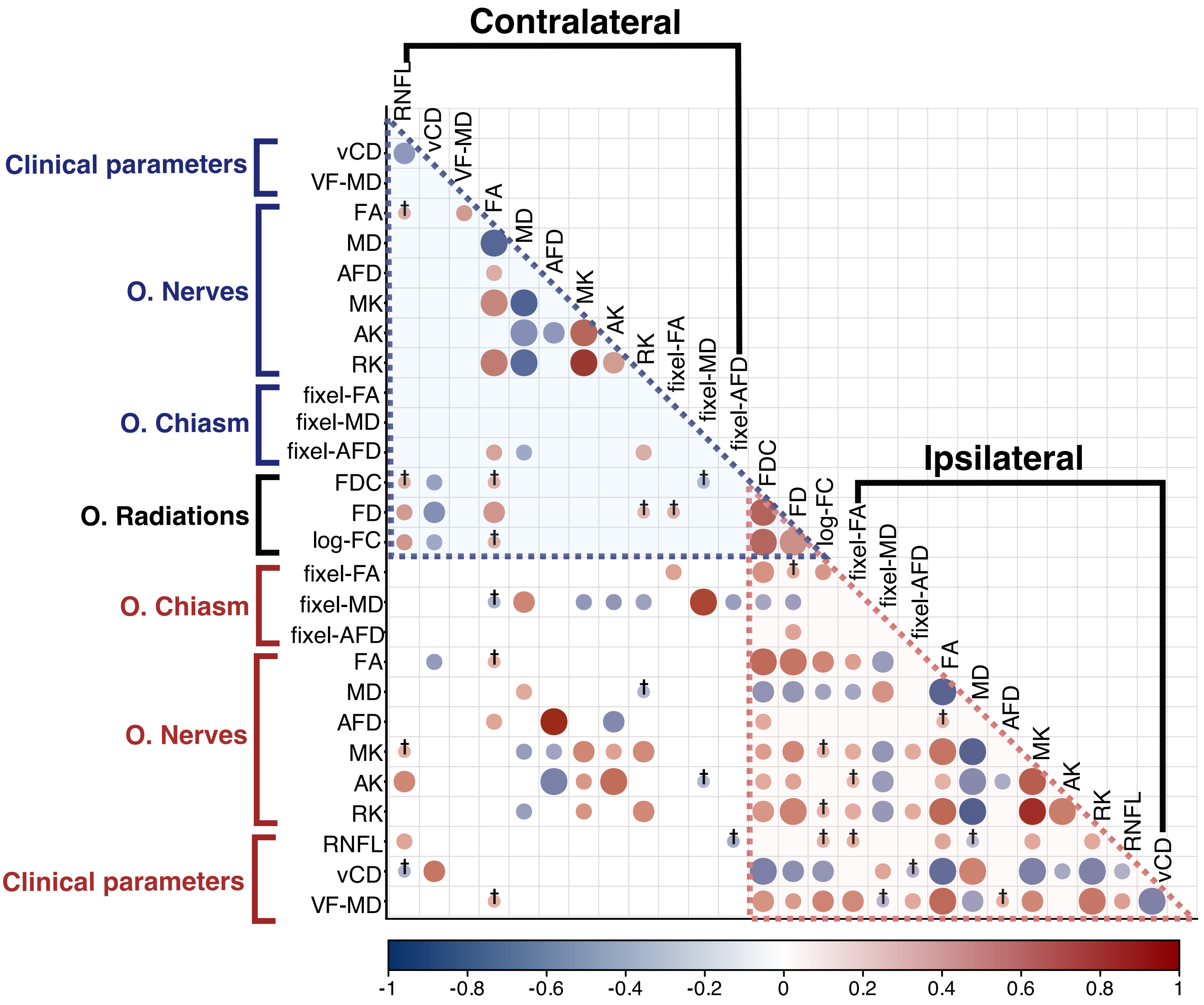

Supplementary Fig S2: Correlation matrix of all diffusion metrics and clinical parameters organized ipsilateral and contralateral to the most affected eyes. Significant correlations are shown as circles (blue = negative, red = positive). Circle diameters reflect the significance of the correlation ($p<0.05$), and crosses indicate trends ($0.05<p<0.1$). The average diffusion metrics of both hemispheres is used for the optic radiations.
